# eMental Health Literacy and Psychological Distress as Predictors of Barriers to Mental Health Services

**DOI:** 10.1093/geroni/igab046.3136

**Published:** 2021-12-17

**Authors:** Eve Root, Grace Caskie

**Affiliations:** 1 Lehigh University, Philadelphia, Pennsylvania, United States; 2 Lehigh University, Bethlehem, Pennsylvania, United States

## Abstract

Since the COVID-19 pandemic, psychologists have begun to rely heavily on technology to provide mental health information and services (APA, 2020). As the older adult population increases, the number of older adults in need of mental health services also increases; however, little is known about the way older adults might utilize technology to inform mental health-related decisions. This study expands on the construct of eHealth Literacy by examining eMental Health Literacy, which is defined as the degree to which individuals seek, find, understand, and appraise basic mental health information and services online that are needed to inform mental health-related decisions. A sample of 244 older adults (M=68.34, range=65-82 years) were recruited online through Amazon Mechanical Turk. A structural equation model was estimated specifying eMental Health Literacy and psychological distress as predictors of extrinsic and intrinsic barriers to mental health services. After adding three correlated errors, the model achieved good fit (χ2(110)=329.20, p<.001, SRMR=.08, CFI=.93, TLI=.91, GFI=.86, RMSEA=.09). All indicators were significantly related to their latent construct (p<.001). The results indicated that, controlling for psychological distress, higher eMental health literacy was significantly related to fewer reported intrinsic (b=-.386, p<.001) and extrinsic barriers (b=-.315, p<.001) to mental health services. Higher distress was also significantly related to more intrinsic (b=.537, p<.001) and extrinsic barriers (b=.645, p<.001) to mental health services. These findings suggest that, as we move towards a more digital world, eMental health literacy could play a significant role in the way older adults navigate through the mental healthcare system.

